# Computed tomography-guided oxygen–ozone injection for the treatment of lumbar disc herniation complicated with lumbar spinal stenosis

**DOI:** 10.4103/mgr.MEDGASRES-D-25-00121

**Published:** 2026-01-06

**Authors:** Jianchong Chen, Zengjie Song, Keya Zheng, Zhichuan Yao, Mengli Wang, Guoming Li, Jinjin Xu, Yufang Gu, Shuo Deng, Qinqin Chen, Yun Xu

**Affiliations:** 1 Center for Rehabilitation Medicine, Department of Pain Management, Zhejiang Provincial People’s Hospital, Affiliated People’s Hospital, Hangzhou Medical College, Hangzhou, Zhejiang Province, China; 2 Department of Rehabilitation, Cixi Hospital of Traditional Chinese Medicine, Cixi Integrated Traditional Chinese and Western Medicine Health Group, Ningbo, Zhejiang Province, China; 3 Department of Pain, the First People’s Hospital of Jiashan County, Zhejiang Province (Jiaxing University Affiliated Jiashan Hospital), Jiaxing, Zhejiang Province, China

**Keywords:** anti-inflammatory, caudal epidural steroid injection, CT-guided, lumbar disc herniation, lumbar spinal stenosis, oxidative ablation, oxygen–ozone injection, ozone therapy, minimally invasive spinal surgery, pain

## Abstract

Lumbar disc herniation complicated by lumbar spinal stenosis is a common degenerative condition in spinal surgery, particularly among middle-aged and elderly individuals. Conservative treatments or open surgery are commonly used but often have limited efficacy or significant risks, especially in older patients. Oxygen–ozone therapy, known for its mechanical decompression, anti-inflammatory, analgesic, and neuroprotective effects, is gaining attention as a minimally invasive treatment for lumbar disc herniation, offering an alternative to traditional treatments. Therefore, this study aimed to evaluate the clinical therapeutic effect of computed tomography-guided percutaneous oxygen–ozone injection on lumbar disc herniation complicated by lumbar spinal stenosis. This retrospective study analyzed the clinical outcomes of 47 patients with lumbar disc herniation complicated by lumbar spinal stenosis who were treated between September 2023 and February 2024. Patients were divided into two groups: the ozone group received computed tomography-guided percutaneous oxygen–ozone injection (*n* = 25), and the caudal epidural steroid injection group underwent ultrasound-guided (*n* = 22). Pain relief and functional outcomes were assessed preoperatively and at 1 day, 1 month, 3 months, and 6 months postoperatively using the visual analog scale, and Oswestry Disability Index and modified MacNab criteria. Both groups showed significant reduction in visual analog scale and Oswestry Disability Index at 1 day and 1 month postoperatively compared with preoperatively (*P* < 0.05), with the ozone group demonstrating more pronounced improvements than the caudal epidural steroid injection group. At 3 months, although further improvement was observed in both groups, the differences in visual analog scale and Oswestry Disability Index between the two groups were not statistically significant (*P* > 0.05). By 6 months, the ozone group showed significantly greater improvements than the caudal epidural steroid injection group (*P* < 0.05). The total effective rate based on modified MacNab criteria increased over time in both groups. Although the ozone group exhibited a slightly higher rate, the difference was not statistically significant (*P* > 0.05). Computed tomography-guided oxygen–ozone injection provides sustained pain relief and functional recovery in lumbar disc herniation with lumbar spinal stenosis, demonstrating superior long-term efficacy to epidural steroids.

## Graphical abstract

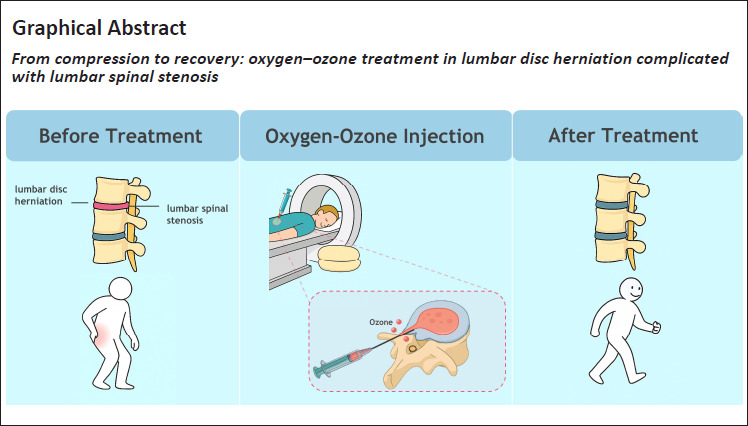

## Introduction

Lumbar disc herniation (LDH) is a common degenerative disease in spinal surgery, most commonly seen in middle-aged and elderly people, usually caused by the nucleus pulposus of the intervertebral disc broken through the annulus fibrosus and then compressing the neighboring neural tissues. The primary clinical manifestations include sciatica, low back pain and cauda equina syndrome.^1^,^2^ In addition, LDH is also associated with cumulative injury, pregnancy, genetic factors, and congenital developmental abnormalities. Patients with LDH alone can usually have significant symptomatic relief through conservative treatment, but those who are combined with stenosis that creates compression often have poor outcomes.^3^,^4^ Lumbar spinal stenosis (LSS) is the leading cause of spinal surgery in individuals over the age of 65 years and is a common source of motor dysfunction in the elderly. It is primarily characterized by narrowing of the spinal canal or intervertebral foramina, resulting in compression of the spinal cord or nerve roots.^5^,^6^,^7^ Historically, open decompressive surgery was the mainstay of treatment for this type of disease. However, in elderly patients, who often present with osteoporosis, degenerative changes, and complex spinal lesions, open procedures can cause significant disruption of the posterior spinal elements. This may lead to complications such as adjacent segment degeneration, epidural adhesions, scarring, and secondary neuropathic pain.^8^,^9^ Conservative treatment options for LSS include physical therapy, pharmacologic interventions, and interventional pain management techniques such as epidural injections.^10^,^11^,^12^,^13^,^14^ Physical rehabilitation focuses on improving core strength and lumbar spine stability to enhance mobility.^15^ Pharmacologic agents such as nonsteroidal anti-inflammatory drugs and calcium channel blockers help alleviate inflammation and pain.^16^,^17^ Additional modalities, including thermal therapy, lumbar traction, and ultrasound, aim to reduce muscular tension and spinal pressure. Epidural injections of glucocorticoids and local anesthetics are also commonly used to reduce inflammation and edema surrounding the nerve roots.^18^ These non-surgical, conservative treatments can relieve the pain caused by LSS, but conservative treatments need professional guidance to avoid further damage to the lumbar spine caused by excessive exercise and need to be adhered to by the patient for a long time. Moreover, pharmacologic treatments carry potential risks such as gastrointestinal irritation and renal impairment. Although epidural injection of glucocorticoids is effective in reducing inflammation and relieving pain, it may also result in adverse effects, such as osteoporosis and neurotoxicity.^19^

The narrowing of the intervertebral foramina in patients with LSS, leading to nerve compression, is caused by a variety of factors, such as osteoarticular degeneration, cartilage degeneration, intervertebral disc degeneration, and trauma. In patients with LSS, the combined effects of multiple factors, such as degenerative changes in intervertebral structures, abnormal neuronal proliferation, and infections, lead to nerve root compression, which triggers symptoms such as pain and numbness.^20^ During this process, excessive accumulation of free radicals triggers oxidative stress, which induces the release of inflammatory cytokines such as interleukin-1β and tumor necrosis factor-α, leading to neuronal damage and dysfunction. The resulting inflammation and nerve compression further compromise local blood supply, causing ischemia and metabolic disturbances, which in turn exacerbate oxidative stress and perpetuate neural injury.^21^,^22^,^23^ At the same time, the inflammatory process also increases vascular permeability and releases substances such as histamine, which further impairs vascular endothelial function, exacerbating LSS.^24^

Ozone disc nucleolysis and percutaneous disc decompression mediated by laser, radiofrequency, or thermal coagulation are the recommended minimally invasive treatment modalities. The principle of most of these methods is based on reducing the disc volume and alleviating the mechanical compression on the nerve roots, thus relieving the symptoms. Among them, the use of ozone therapy in the treatment of LDH has gradually increased and achieved better clinical results.^25^,^26^,^27^ It has been found that intradiscal oxygen–ozone infiltration not only reduces the requirement for conventional surgery and reduces the hospitalization time and cost of patients but also relieves disc-induced pain through ozone dissolution and has a fast recovery.^26^,^28^,^29^ Studies have shown that ozone not only reduces the volume of the nucleus pulposus, but also has an anti-inflammatory and neuroprotective effect, and has shown a better effect in relieving the symptoms of patients.^30^,^31^ Low concentrations of ozone exhibit analgesic and anti-inflammatory effects and can reduce the expression of serum interleukin-6, immunoglobulin G, and immunoglobulin M, effectively treating LDH.^27^ In addition, ozone can inhibit the synthesis and release of proinflammatory cytokines, prostaglandin E2, and bradykinin, as well as stimulate the release of anti-inflammatory cytokines, exerting an effective analgesic and anti-inflammatory effect.^32^,^33^ Studies have shown that ozone can reduce inflammation by activating the AMP-activated protein kinase up-regulation of the growth arrest-specific gene 6/MER proto-oncogene tyrosine kinase/suppressor of cytokine signaling 3 signaling pathway.^34^,^35^ While research on ozone therapy for LDH continues to expand,^36^,^37^ its therapeutic applications in LSS require further investigation.

Considering the limitations of both conservative therapies and traditional open surgical interventions—especially in elderly patients with LDH complicated by LSS—there is a growing demand for safe, effective, and minimally invasive alternatives. Among these, computed tomography (CT)-guided intradiscal oxygen–ozone injection has emerged as a promising technique due to its ability to precisely target the lesion site while minimizing collateral tissue damage. However, its clinical application in patients with LDH combined with LSS remains insufficiently studied. Therefore, this study aimed to evaluate the clinical efficacy of CT-guided oxygen–ozone injection in the treatment of LDH combined with LSS, in comparison with conventional caudal epidural steroid injection (CESI) to explore its application value in relieving patients’ pain and improving their quality of life.

## Methods

### Subjects

This retrospective study included 47 consecutive patients with LDH complicated by LSS, who were admitted to the Department of Rehabilitation Management of Cixi Hospital of Traditional Chinese Medicine between September 2023 and February 2024 and met the inclusion criteria, and were enrolled as study subjects. All included patients were selected consecutively based on predefined inclusion and exclusion criteria, and data collection was conducted using standardized clinical records. Surgical procedures and outcome assessments, including visual analog scale (VAS), Oswestry Disability Index (ODI), and modified MacNab criteria, were performed by the same experienced physician using consistent methods. This study was approved by the Ethics Committee of Cixi Hospital of Traditional Chinese Medicine (approval No. SL2023003, approval date: September 5, 2023), with a waiver of informed consent. The trial was reported in accordance with the STrengthening the Reporting of OBservational studies in Epidemiology (STROBE) guidelines^38^ and performed in strict accordance with the *Declaration of Helsinki*.

Inclusion criteria comprised: (1) presence of low back pain and/or radicular leg pain; (2) magnetic resonance imaging-confirmed LDH with concurrent LSS; (3) persistent pain for ≥ 6 months unresponsive to conservative treatment; and (4) VAS score^39^ ≥ 4.

Exclusion criteria included: (1) prior lumbar nerve block or disc intervention; (2) pre-existing radiculopathy, spinal cord injury, or cauda equina syndrome; (3) severe spinal stenosis with persistent neural ischemia or irreversible neural damage; and (4) systemic comorbidities such as active infection or coagulopathy.

Patients were randomly allocated into two groups: the ozone group (*n* = 25) received CT-guided oxygen–ozone injection therapy, while the CESI group (*n* = 22) underwent ultrasound-guided CESI, with all patients completing at least 6 months of postoperative follow-up. Unlike the CESI group, the ozone group did not receive corticosteroids or anesthetics; instead, oxygen–ozone alone was administered.

### Treatment

#### Ozone group

**Figure 1:**
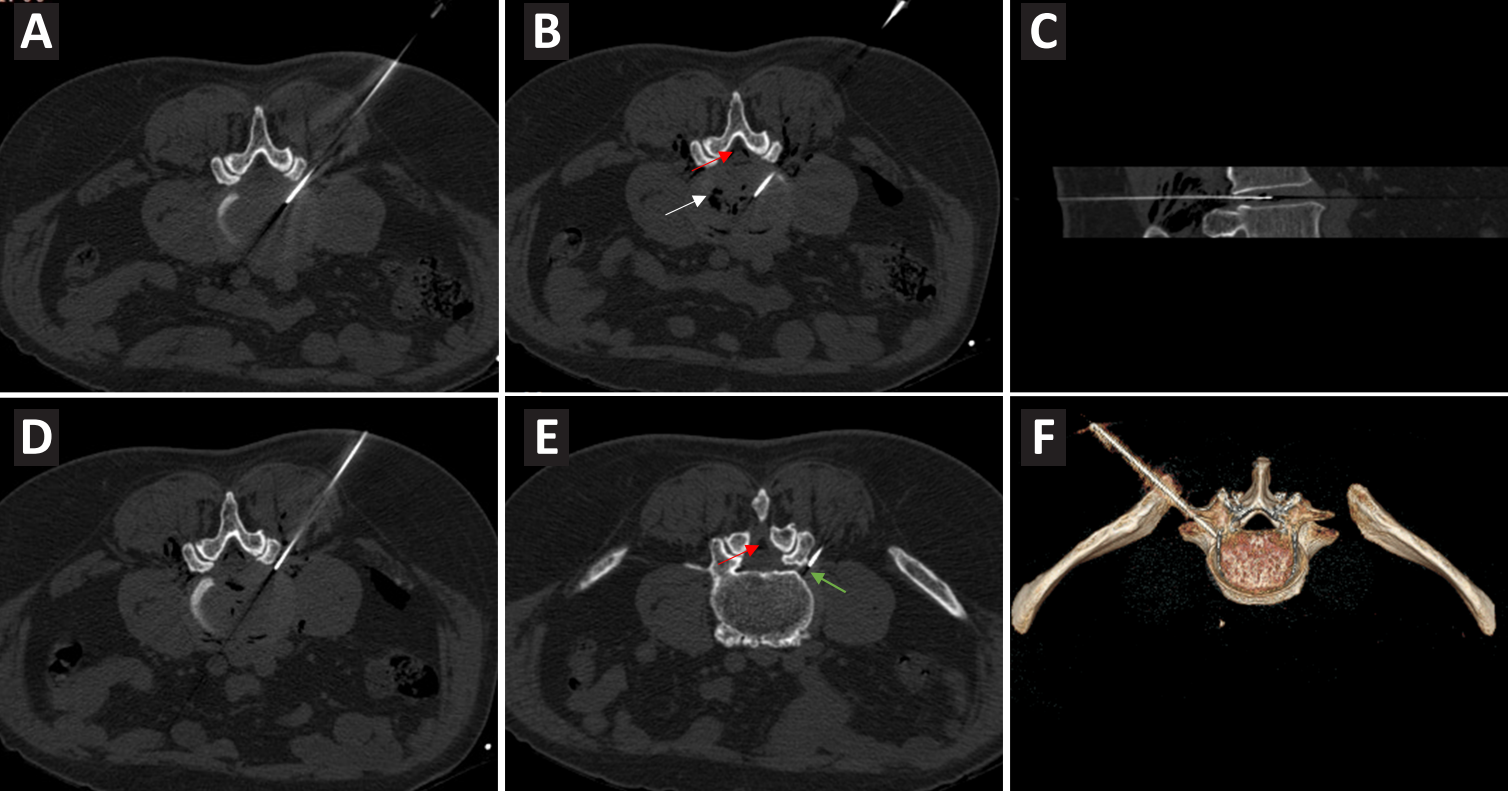
CT-guided injection of medical oxygen–ozone for the treatment of lumbar disc herniation combined with spinal canal stenosis. (A) The needle tip of the syringe is positioned at the posterior center of the affected intervertebral disc. (B) Injection of oxygen–ozone into the center of the affected intervertebral disc. (C) Sagittal view showing the needle tip positioned within the affected intervertebral disc. (D) The needle tip is withdrawn to the area near the intervertebral foramen. (E) Injection of oxygen–ozone near the intervertebral foramen. (F) Three-dimensional reconstruction image during the injection process. White arrow indicates visible gas; red arrow indicates gas within the spinal canal; green arrow indicates visible gas. CT: Computed tomography; LDH: lumbar disc herniation; LSS: lumbar spinal stenosis.

All patients in the ozone group were placed in a prone position. Before the operation, the diseased intervertebral space was scanned under CT (Siemens, Erlangen, Germany), and the distance from the puncture point to the intervertebral disc, the angle of needle insertion, and the depth of needle insertion were determined, and then the puncture insertion point was marked on the skin. Local anesthesia was administered with 2% lidocaine (Shanghai Zhaohui Pharmaceutical Co. Ltd., Shanghai, China), and a 21G puncture needle was used to puncture the diseased intervertebral disc under CT guidance so that the tip of the needle was located in the center of the intervertebral space in a posterior position (**Figure 1A**). Then, a disposable sterile syringe was used to inject 40 µg/mL of oxygen–ozone from the ozone generator (Ozomed Smartline medical ozone therapy instrument, Hänsler Medical GmbH, Bruchsal, Germany) into the intervertebral disc (**Figure 1B** and **C**), and the amount injected was determined by the patient’s response, usually 12–20 mL. Then the needle was withdrawn to the vicinity of the intervertebral foramen (**Figure 1D–F**), and 3–5 mL of oxygen–ozone at 25 µg/mL was injected into both the paravertebral space and spinal canal. The patient was then required to lie on a hard bed for 2 hours postoperatively before resuming normal activities.

#### CESI group

All CESI patients were similarly placed in the prone position. The probe was placed transversely on the sacrum to show the sacral angle on both sides of the cross-section; the probe was rotated 90° to obtain a longitudinal sagittal view of the sacral fissure, and the sacral fissure was located in the center of the screen. Aiming at the sacral fissure under ultrasound (M7 Series color Doppler ultrasound system, Shenzhen Myriad Medical Instrument Company, Shenzhen, China) guidance, a puncture needle is passed through the sacrococcygeal ligament, and 20 mL of pain compound (1 mL of 5 mg/mL betamethasone (Merck, Kenilworth, NJ, USA) + 3 mL of 2% of lidocaine + 16 mL of normal saline) is injected after retracting the blood- or cerebrospinal fluid-free. After the operation, the patient also needs to lie absolutely on a hard board bed for 2 hours, after which he can move normally.

### Outcomes

All patients were followed up for 6 months postoperatively. Pain intensity was assessed using VAS preoperatively, and at 1 day, 1 month, 3 months, and 6 months postoperatively. Functional improvement was assessed using ODI at the same time points. Clinical efficacy was evaluated using the modified MacNab criteria.^40^ Treatment outcomes were categorized into three levels: ineffective, effective, and obvious effect. Ineffective was defined as no significant improvement in clinical symptoms or signs, no meaningful reduction in VAS score, an improvement rate in ODI of less than 30% compared to baseline, and a continued need for medication or surgical intervention. Effective was defined as partial improvement in symptoms, reduced medication requirement, a VAS score reduction of 2–3 points, and an ODI improvement rate between 30% and 70%. Obvious effect was defined as marked relief of clinical symptoms and signs, no need for further treatment, a VAS score reduction of 4 points or more, and an ODI improvement rate of 70% or greater. The total effective rate was calculated as follows: (number of cases with obvious effect + number of effective cases)/total number of cases × 100%.

### Statistical analysis

All analyses were performed using SPSS 18.0 (SPSS Inc., Chicago, IL, USA). Continuous variables are presented as the mean ± standard deviation (SD), while categorical data are expressed as number (percentage). Between-group comparisons were assessed using independent *t*-test for continuous variables and chi-square test for categorical parameters. Statistical significance was defined as two-tailed *P* < 0.05.

## Results

### Basic information of patients with lumbar disc herniation combined with lumbar spinal stenosis

A total of 47 patients were enrolled in this study, including 22 patients in CESI group and 25 patients in oxygen group. The CESI group consisted of 8 males (36%) and 14 females (64%). The ozone group included 10 males (40%) and 15 females (60%). These differences in sex distribution were not statistically significant (*P* > 0.05). The patients’ stenosis sites were mainly in L3/4, L4/5, and L5/S1, and there was no statistical difference between the two groups. The site of pain was mainly in the lumbar region, with lower limb pain, and there was no statistically significant difference between the two groups (*P* > 0.05; **Table 1**).

**Table 1:** Baseline information in patients with LDH combined with LSS of two groups

	Caudal epidural steroid injection group (*n* = 22)	Ozone group (*n* = 25)	*P*-value
Age (year)	62.136±11.235	61.960±11.956	0.61
Sex			0.798
Male	8 (36)	10 (40)	
Female	14 (64)	15 (60)	
Restricted segment			0.557
L3/4	1	1	
L4/5	12	17	
L5/S1	5	2	
L4/5 & L5/S1	4	5	
Pain area			0.994
Low back pain without lower extremity pain	5	6	
Low back pain with left	4	5	
lower extremity pain			
Low back pain with right	6	6	
lower extremity pain			
Low back pain with bilateral	7	8	
lower extremity pain			

Continuous variables are presented as the mean ± SD, and were analyzed by independent *t*-test. Categorical data are expressed as number (percentage), and were analyzed by chi-square test. LDH: Lumbar disc herniation; LSS: lumbar spinal stenosis.

### Pain intensity and functional improvement of CT-guided oxygen–ozone injection

To evaluate whether CT-guided oxygen–ozone injection provides superior pain relief and functional improvement compared to CESI, we compared VAS and ODI between the two groups at different postoperative time points. Preoperatively, there were no significant differences between the ozone group and the CESI group in either VAS or ODI scores (*P* > 0.05), indicating baseline comparability. Following treatment, both groups showed significant reductions in VAS and ODI scores compared to preoperative values (*P* < 0.05), demonstrating effective symptom relief. At 1 day post-treatment, the CESI group exhibited significantly lower VAS and ODI scores compared to the ozone group (both *P* < 0.05), indicating more rapid early symptom relief. This short-term advantage was partially maintained at 1 month, where the CESI group continued to have significantly lower ODI scores (*P* < 0.05), although no statistically significant difference was observed in VAS scores between the two groups (*P* > 0.05). By 3 and 6 months post-treatment, the trend reversed: the ozone group demonstrated significantly lower VAS scores at both 3 months and 6 months (both *P* < 0.05), and lower ODI scores at 6 months (12.46 ± 6.41 *vs*. 16.52 ± 6.99, *P* < 0.05), indicating a more sustained and durable therapeutic effect (**Table 2**). These findings suggest that while CESI may offer faster early symptom control, CT-guided oxygen–ozone injection provides more durable and effective long-term benefits in patients with LDH combined with LSS. No adverse reactions were observed in both groups of patients during surgery and at follow-up.

**Table 2:** VAS and ODI scores of patients with LDH combined with LSS in both groups

	Caudal epidural steroid injection group (*n* = 22)	Ozone group (*n* = 25)	*t*-value	*P*-value
VAS				
Preoperative	5.818±1.094	6.028±1.191	0.658	0.515
1 day postoperatively	2.840±0.533	3.870±0.847	4.909	< 0.001
1 month postoperatively	3.309±1.211	3.548±0.876	0.781	0.439
3 month postoperatively	3.559±0.624	2.692±0.535	5.129	< 0.001
6 month postoperatively	3.123±0.578	1.864±0.579	7.444	< 0.001
ODI				
Preoperative	44.182±11.868	46.156±9.715	0.627	0.534
1 day postoperative	14.523±6.587	31.460±8.787	7.392	< 0.001
1 month postoperatively	21.068±6.118	27.220±8.673	2.773	0.008
3 month postoperatively	23.136±4.851	21.940±8.951	0.509	0.579
6 month postoperatively	16.523±6.987	12.456±6.405	2.082	0.043

Data are expressed as the mean ± SD, and were analyzed by independent *t*-test. LDH: Lumbar disc herniation; LSS: lumbar spinal stenosis; ODI: Oswestry Disability Index; VAS: visual analog scale.

### Clinical efficacy of CT-guided oxygen–ozone injection

To determine whether CT-guided oxygen–ozone injection provides superior clinical efficacy compared to CESI in patients with LDH combined with LSS, we analyzed the response rates at multiple postoperative time points (**Table 3**). At 1 day post-treatment, the CESI group demonstrated a higher overall response rate than the ozone group, suggesting more rapid short-term symptom relief; however, the difference was not statistically significant (*P* > 0.05). By 1 month, the overall response rates in the two groups were similar (*P* > 0.05). At 3 months, the ozone group exhibited a higher overall response rate and a greater proportion of obvious effect cases, but again, the difference did not reach statistical significance (*P* > 0.05). By the 6-month follow-up, the ozone group continued to show a slightly higher overall response rate, along with more “obvious effect” cases and fewer poor-effect cases. Nevertheless, the difference between the two groups remained statistically non-significant (*P* > 0.05). These findings suggest that although CT-guided oxygen–ozone injection may offer a trend toward more sustained clinical benefit over time, particularly beyond 3 months post-treatment, it did not demonstrate a statistically significant advantage over CESI in terms of overall response rate during the 6-month follow-up period.

**Table 3:** Clinical efficacy of patients with LDH combined with LSS in two groups

	Caudal epidural steroid injection group (*n* = 22)	Ozone group (*n* = 25)	χ^2^-value	*P*-value
1 day postoperative			2.59	0.27
Obvious effect	5 (23)	3 (12)		
Effective	14 (64)	14 (56)		
Overall response	19 (87)	17 (68)		
1 month postoperatively			0.07	0.97
Obvious effect	3 (14)	4 (16)		
Effective	13 (59)	14 (56)		
Overall response	16 (73)	18 (70)		
3 months postoperatively			1.71	0.43
Obvious effect	3 (14)	5 (20)		
Effective	12 (55)	16 (64)		
Overall response	15 (68)	21 (84)		
6 months postoperatively			0.54	0.77
Obvious effect	4 (18)	6 (24)		
Effective	15 (68)	17 (68)		
Overall response	19 (86)	23 (92)		

Data are expressed as number (percentage), and were analyzed by chi-square test. LDH: Lumbar disc herniation; LSS: lumbar spinal stenosis.

## Discussion

This study retrospectively investigated the clinical efficacy of CT-guided ozone injection in the treatment of patients with LDH combined with LSS, in comparison with ultrasound-guided CESI. In recent years, a large number of studies at home and abroad have confirmed that the treatment of lumbar intervertebral disc herniation with ozone injection is minimally invasive, economical, simple, and safe.^25^,^41^,^42^,^43^,^44^ Compared with traditional open surgery, its efficacy is not inferior, the recovery time is short and the risk is lower. This therapeutic approach also yields excellent clinical outcomes for patients presenting with LDH complicated by LSS. In our study, both groups showed high overall effective rates at the 6-month follow-up; however, the difference was not statistically significant. This suggests that CT-guided oxygen–ozone therapy may offer similar clinical benefits to conventional CESI in relieving symptoms of LDH combined with LSS. Although no significant difference in total effective rate was observed, patients in the ozone group exhibited more pronounced improvements in pain and functional scores at later follow-ups, indicating potential advantages in long-term symptom control and quality of life.

The innovation of this study lies in the integration of CT-guided precision positioning with ozone-based minimally invasive therapy for LDH complicated by LSS. While traditional ozone therapy relies on oxidative ablation to reduce intradiscal pressure and relieve nerve root compression, it often suffers from limitations in anatomical accuracy and gas distribution.^26^,^45^ In this study, the use of CT guidance significantly improved needle placement accuracy and allowed precise control of ozone diffusion within the target disc area, thereby enhancing therapeutic efficacy and safety. Based on the precise positioning of the CT, we first ensured that the tip of the puncture needle accurately reached the target point of the stenotic disc, and then determined the amount of ozone injected according to the patient’s response, which was usually 12–20 mL of medical ozone at 40 µg/mL. By repeatedly inserting or withdrawing the needle, we could increase the diffusion of ozone in the intervertebral disc to enhance the contact between the ozone and disc tissues, and then give full play to the oxidative-ablative effect of the ozone.^46^ In addition, a low concentration of ozone (25 µg/mL, 3–5 mL) was injected into the intervertebral foramen and paravertebral space, and compound betamethasone injection was injected into the posterior margin of the vertebral body to strengthen the anti-inflammatory and analgesic effects, which is equivalent to the “local closure” of the nerve root, to better play the role of ozone minimally invasive therapy.^47^ Moreover, CT guidance technology allows physicians to accurately observe the dispersion of ozone gas, ensuring that the ozone is widely and evenly distributed to maximize its therapeutic effect. Compared with traditional C-arm X-ray guidance, CT guidance has higher accuracy, especially in complex cases, and can more clearly show the characteristics of intervertebral disc rupture and the distribution of gas, thus improving the safety and effectiveness of treatment.

In the present study, no complications or adverse events were reported during the follow-up period. Previous literature has documented relatively few complications related to ozone therapy for LDH.^42^,^48^,^49^ In most cases, only mild side effects such as insomnia, pruritus, and other general symptoms have been observed. However, in rare instances, serious adverse reactions may occur, including infection, neurological injury, and cerebrovascular accidents. Ginanneschi et al.^50^ reported a case in which a patient developed numbness along the anterolateral aspect of the left leg and hypoesthesia on the dorsum of the left foot following ozone therapy, which was attributed to spinal nerve injury. This suggests that ozone therapy has the potential to damage peripheral nerves, particularly if the procedure is performed improperly, as direct injury to nerve structures may occur during injection. In addition, a study has reported cases of dense adhesions following ozone therapy, especially firm connections between the nerve root, the dural sac, and/or herniated disc fragments.^51^ These adhesions are often difficult to separate, leading to nerve root retraction and the formation of a tough fibrotic capsule, thereby complicating surgical dissection. The formation of such adhesions may result from the direct irritative or cytotoxic effects of ozone on neural structures, triggering local inflammation and excessive fibrosis during tissue repair. Factors such as ozone dosage, injection frequency, and individual tissue responses may contribute to adhesion development. Although injections near the intervertebral foramina and within the spinal canal were performed in this study, the absence of neurological injuries may be attributed to low doses and CT guidance precision. As with any invasive procedure, attention must be paid to potential complications, particularly infection. There are reports of fulminant sepsis^52^ and pyogenic spondylitis^53^ caused by ozone therapy, possibly due to inadequate aseptic technique or immunosuppressive effects of ozone on local tissues, which may facilitate bacterial invasion and proliferation. Therefore, strict adherence to aseptic principles during the procedure is essential.

This study has shown significant efficacy in clinical application, but we are aware of some of its limitations. First, due to the small sample size and short follow-up period, it is difficult for us to fully assess the long-term effects of ozone therapy and its recurrence rate. To address this issue, future studies should expand the sample size and extend the follow-up period to more accurately assess the durability and stability of the treatment effect. The efficacy of ozone therapy is closely related to the technique of manipulation. The skill level of the practitioner in determining key operations such as puncture points, angles, and depths is critical to ensure the accuracy and effectiveness of the treatment. We therefore propose to strengthen the professional training of practitioners to enhance the precision of the operation.

To further enhance the therapeutic effect, future studies should also consider optimizing the injection method and concentration of ozone. In particular, for patients with spinal stenosis combined with LDH, the combination of ozone therapy with other minimally invasive techniques such as radiofrequency ablation or intervertebral foraminoscopy can be considered with the aim of obtaining better clinical efficacy. With these measures, we expect to overcome the current limitations and further enhance the clinical application value of ozone therapy in future studies. Moreover, given the standardized CT-guided protocol and favorable safety profile observed in this study, the technique may be applicable across a broad range of clinical settings. This suggests that CT-guided oxygen–ozone injection holds promise for widespread clinical adoption, especially in elderly patients with LDH complicated with LSS who are poor candidates for open surgery. However, multicenter randomized controlled trials with larger sample sizes are needed to validate its generalizability and long-term efficacy.

## Data Availability

*The datasets generated during and/or analyzed during the current study are available from the corresponding author on reasonable request*.
